# Cardiac acoustic biomarkers as surrogate markers to diagnose the phenotypes of pulmonary hypertension: an exploratory study

**DOI:** 10.1007/s00380-021-01943-7

**Published:** 2021-10-01

**Authors:** Nobuhide Yamakawa, Norihiko Kotooka, Tomoyuki Kato, Tatsuhiko Kuroda, Koichi Node

**Affiliations:** 1 Healthcare R&D Center, Asahi Kasei Corporation, 1-1-2 Yurakucho, Chiyoda-ku, Tokyo, 100-0006 Japan; 2 Department of Cardiovascular Medicine, Saga University, 5-1-1 Nabeshima, Saga, Saga 849-8501 Japan

**Keywords:** Cardiac acoustic biomarkers, Echocardiography, Phenotype, Pulmonary hypertension, Right heart catheterization

## Abstract

Pulmonary hypertension (PH) is commonly associated with left heart disease. In this retrospective study, using the database of a clinical study conducted between January 2008 and July 2008, the phenotypes of PH were classified using non-invasive cardiac acoustic biomarkers (CABs) and compared with classification by echocardiography. Records with same-day measurement of acoustic cardiography and right heart catheterization (RHC) parameters were included; cases with congenital heart disease were excluded. Using the RHC measurements, PH was classified as pre-capillary PH (Prec-PH), isolated post-capillary PH (Ipc-PH), and combined pre-capillary and post-capillary PH (Cpc-PH). The first, second, third, and fourth heart sounds (S1, S2, S3, and S4) were quantified as CABs (intensity, complexity, and strength). Forty subjects were selected: 5 had Prec-PH, 5 had Ipc-PH, 8 had Cpc-PH, and 22 had No-PH. CABs were significantly correlated with RHC measurements, with significant differences among phenotypes. Phenotype classification was performed using various CABs, and the diagnostic performance as assessed by the area under the receiver operating characteristic curve was 0.674–0.720 for Prec-PH, 0.657–0.807 for Ipc-PH, and 0.742 for Cpc-PH. High negative and low positive predictive values for phenotype identification were observed. CABs may provide an ambulatory measurement method with home-monitoring friendliness which is more convenient than standard examinations to identify presence of PH and its phenotypes.

## Introduction

Pulmonary hypertension (PH) is commonly associated with left heart disease and categorized as PH due to left heart disease (PH-LHD) [1]. The majority of PH-LHD patients are reported to have heart failure (HF), with both reduced and preserved ejection fraction [2], which is a well-known pathological condition for functional and structural impairment in the heart [3]. The structure and hemodynamic status of the pulmonary circulation are largely affected by both pulmonary and cardiac failure, possibly leading to a poor prognosis due to remodeling in the right ventricle, as well as in the pulmonary artery and veins [4]. Diagnostic and therapeutic strategies for PH-LHD are, however, not yet established [5]; therefore, its management is one of the clinical challenges for both PH and HF experts.

PH is mainly characterized by increased pulmonary artery pressure (PAP) on right heart catheterization (RHC) [6]. RHC provides precise information not only for PH diagnosis, but also to differentiate PH phenotypes [pre-capillary PH (Prec-PH), isolated post-capillary PH (Ipc-PH), and combined pre- and post-capillary PH (Cpc-PH)] when other hemodynamic parameters, such as pulmonary artery wedge pressure (PAWP) and pulmonary vascular resistance (PVR), are available [7]. PH-LHD is categorized as post-capillary PH, and in the PH with HF population, 14% of systolic and 12% of diastolic HF cases were reported to have Cpc-PH, and the rest had Ipc-PH [8]. Since the pathophysiology and prognosis of Cpc-PH are distinct from those of Ipc-PH, differentiation of these two phenotypes by measuring PVR is clinically important [8]. Only PAP has been a target of continuous monitoring for HF patients by an implantable device to decrease the risk of re-hospitalization [9], but no other hemodynamic parameters to classify PH can be measured by such monitoring. Although heart and pulmonary function can be evaluated by measurement of PAP, providing much clinically useful information, invasive procedures are needed, which involve risk to the patients [10, 11].

To estimate PAP for PH diagnosis without invasive technology, surrogate methods using echocardiography measurements have been attempted [12, 13]. Moreover, validation studies to distinguish the phenotypes, pre-capillary versus post-capillary PH, using echocardiographic assessment have been performed [14–16]. Although echocardiography is a non-invasive and inexpensive method to estimate PAP and other hemodynamic parameters, the correlation and the specificity in the previous reports were insufficient for PH diagnosis and phenotyping [17–20].

Acoustic cardiography is a technology for quantifying heart and pulmonary status by non-invasive measurement of cardiohemic vibrations (cardiac cavities, valves, and blood) with simultaneously recorded electrocardiograms (ECGs) and automatic calculation of cardiac acoustic biomarkers (CABs) [21]. Non-invasive estimation of PAP using CABs has been previously reported [22, 23]. The relationship between the second heart sound measured by acoustic cardiography and PAP measured by RHC was evaluated cross-sectionally in a pre-capillary PH population, e.g., pulmonary artery hypertension (PAH), but a comprehensive evaluation of patients with post-capillary PH or PH-LHD and its specific phenotypes, Ipc- and Cpc-PH, has not been performed.

In this analysis, the correlations between measurement parameters of RHC and CABs were evaluated, and the diagnostic accuracy for different phenotypes of PH was evaluated.

## Materials and methods

### Subjects

The present study was based on a retrospective analysis using the database from a study conducted at the University of Utah Health Sciences Center and The Veterans Administration Salt Lake City Health Care System between January 2008 and July 2008. All patients referred for RHC, as well as ICU inpatients with a Swan-Ganz pulmonary artery catheter inserted, in the hospitals were included. Hemodynamically unstable patients, those with a high degree of noise in the vicinity of their chest, e.g. positive pressure ventilation, and those with congenital heart disease (CHD) were excluded.

This study was conducted in compliance with the Declaration of Helsinki. The study protocol was reviewed and approved by the ethics committee of Saga University, Japan. Written, informed consent was obtained from all subjects before measurement of baseline data.

### Diagnosis of pulmonary hypertension

The diagnosis and the phenotypes of PH were classified by mean PAP, PAWP, and PVR by RHC in accordance with the updated clinical classification of PH [7]: Prec-PH (mean PAP > 20 mmHg, PAWP ≤ 15 mmHg, and PVR ≥ 3 Wood Units); Ipc-PH (mean PAP > 20 mmHg, PAWP > 15 mmHg, and PVR < 3 Wood Units; Cpc-PH (mean PAP > 20 mmHg, PAWP > 15 mmHg, and PVR ≥ 3 Wood Units), and No-PH. Since the measurement values of PVR were recorded in dynes s cm^−5^, the threshold of 3 Wood Units was substituted by 240 dynes s cm^−5^.

### Right heart catheterization

RHC was performed with the patients at rest without sedation, and PAP, PAWP, PVR, and cardiac output (CO) were obtained. The CO measured by the Fick method was used in the analysis and normalized by body surface area (BSA) to calculate the cardiac index (CI).

### Cardiac acoustic biomarkers (CABs)

Baseline heart sounds and ECGs were recorded simultaneously by acoustic cardiography [24] before catheterization at rest. A Holter ECG recorder (AUDICOR AM-RT, Inovise Medical Inc, Portland, OR, USA) was used for acoustic cardiography a few hours after RHC. The recorder had three electrodes at the right upper, left upper, and left lower chest areas for electrocardiography, and two other electrodes with an accelerometer were placed at five different locations of the chest wall in the following three combinations: V3 and V4 (apex), V3 and the second left intercostal space (2L), and the third left intercostal space (3L) and the second right intercostal space (2R). Based on the empirical knowledge of auscultation, the data recorded at V4 and 3L sites were used for later analysis to focus on extra heart sounds (S3 and S4) and S2 splitting, respectively. ECG and phonocardiogram (PCG) data for each location were approximately 1.5 min in length.

**Fig. 1:**
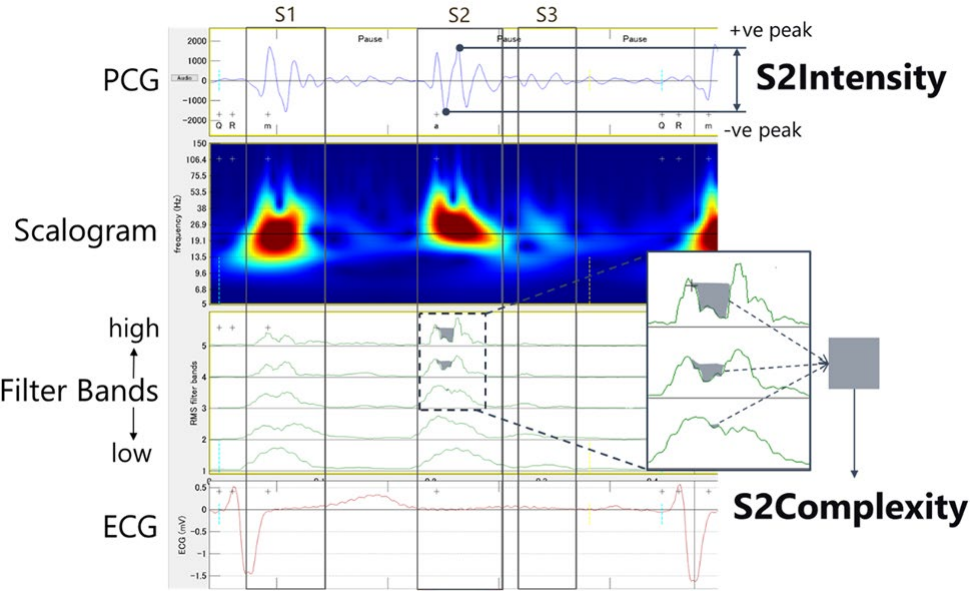
Visual description of SnIntensity and SnComplexity as CABs. In this example, the patient has pre-capillary PH, and CABs shows S2Intensity = 5.4 and S2Complexity = 9.8, which are high in comparison with the control (No-PH) population. The same principle can be applied to the other intensity and complexity values, e.g., S1Intensity and S1Complexity. The scalogram visualizes the changes of frequency components with time and aids users to observe abnormalities in heart sounds. The phonocardiogram (PCG) signal is decomposed into five different frequency components in the filter bands row

The heart sound categories, i.e., first, second, third, and fourth heart sounds (S1, S2, S3, and S4), synchronized with the ECG, were converted into the CABs of intensity, complexity [23], and strength [24] for each heart sound. The calculation methods for intensity and complexity are shown in Fig. 1 as an example of deriving S2Intensity and S2Complexity. SnIntensity quantifies the intensity of each heart sound category, where *n* ranges from one to four, based on peak-to-peak amplitude on the PCG inside the segments of the heart sounds. In Fig. 1, split S2 vibration shows a wide and complex waveform in its filter bands with multiple peaks. The complexity of a waveform is quantified by SnComplexity, where the areas of valleys created by the peaks in high-frequency components inside the segment are summed. S3Strength and S4Strength are probability scores based on acoustic features reflecting the presence of an audible S3 and an audible S4 on standard auscultation. They range from 0 to 10, and values above five indicate the existence of a clinically audible S3 or S4 in a 10-s recording.

### Statistical analysis

Baseline numerical data are shown as means and standard deviation (SD). Categorical data are indicated as numbers and percentages. Characteristics of subjects across phenotypes of PH were compared by ANOVA or the *χ*^2^ test. The correlations between RHC parameters (mean PAP, PAWP, PVR, CI) and CABs (intensity, complexity, and strength) were evaluated by Pearson’s correlation coefficients. Differences in measurement values on RHC and CABs among the 4 groups (Prec-PH, Ipc-PH, Cpc-PH, versus No-PH) were evaluated by *t* test. To estimate the accuracy of differentiating the three PH types by CABs, receiver operating characteristic (ROC) analysis was performed to test sensitivity and specificity with different thresholds, as well as the area under the curve (AUC). The thresholds of each parameter for evaluating accuracy were selected according to the maximum value of sensitivity- (1-specificity). The positive predictive value (PPV) and negative predictive value (NPV) were also calculated to evaluate diagnostic characteristics. All reported *p* values are two tailed, and *p* < 0.05 was taken to indicate significance. All statistical analyses were performed using JMP version 15 (SAS Institute, Cary, NC, USA).

## Results

A total of 40 subjects were selected for this analysis from the database. The number of subjects in each phenotype of PH was as follows: Prec-PH, 5 cases; Ipc-PH, 5 cases; Cpc-PH, 8 cases; and No-PH, 22 cases. There were no significant differences in the subjects’ characteristics among the PH phenotypes (Table 1).

**Table 1:** Subjects’ characteristics

Item		Type of PH				*p*
		Prec-PH (*n* = 5)	Ipc-PH (*n* = 5)	Cpc-PH (*n* = 8)	No-PH (*n* = 22)	
Age	years	55.8 ± 16.7	66.0 ± 12.0	60.8 ± 13.0	52.4 ± 16.5	0.271
BMI	kg/m^2^	27.2 ± 10.3	32.2 ± 9.6	28.1 ± 6.9	28.9 ± 6.0	0.703
Sex	Male (%)	1 (20.0)	3 (60.0)	5 (62.5)	17 (77.3)	0.117
Classification	Inpatient (%)	1 (20.0)	1 (20.0)	5 (62.5)	5 (22.7)	0.196
Current smoker	Yes (%)	0 (0.0)	1 (20.0)	0 (0.0)	0 (0.0)	0.226
NYHA class	0/I/II/III/IV	1/3/1/0/0	1/2/1/0/1	1/0/4/2/1	9/9/2/2/0	0.053
Hypertension	Yes (%)	3 (60.0)	4 (80.0)	5 (62.5)	20 (90.9)	0.230
Diabetes	Yes (%)	1 (20.0)	2 (40.0)	3 (37.5)	6 (27.3)	0.857
Dyslipidemia	Yes (%)	3 (60.0)	4 (80.0)	5 (62.5)	19 (86.4)	0.422
Obstructive sleep apnea	Yes (%)	1 (20.0)	2 (40.0)	3 (37.5)	4 (18.2)	0.614
Coronary artery disease	Yes (%)	1 (20.0)	2 (40.0)	4 (50.0)	8 (36.4)	0.847

Data presented as means ± SD or numbers (%)

*PH* pulmonary hypertension, *Prec-PH* pre-capillary PH, *Ipc-PH* isolated post-capillary PH, *Cpc-PH* combined pre- and post-capillary PH, *NYHA* New York Heart Association function class

*p* value represents difference between types of PH

The correlations of the RHC parameters and CABs are presented in Table 2. Significant correlations were observed between mean PAP and S2Complexity (V4) or S3Strength (V4), PAWP and S3Intensity (V4) or S3Strength (3L, V4), PVR and S2Complexity (3L, V4), and CI and S1Intensity (3L) or S1Complexity (V4). RHC parameters and CABs of the PH phenotypes are shown in Tables 3 and 4. All RHC parameters were significantly different between Cpc-PH and No-PH. Mean PAP and PVR in Prec-PH and mean PAP and PAWP in Ipc-PH were significantly higher than in No-PH (Table 3). S2Intensity, S2Complexity in Prec-PH, and S2, S3, and S4Intensity in Ipc-PH were significantly higher than in No-PH (both 3L, V4, Table 4). S3Strength was significantly higher in Cpc-PH than in No-PH.

**Table 2:** Correlations between RHC parameters and CABs

Parameter		Measurement site			
RHC	CABs	3L		V4	
		*r*	*p*	*r*	*p*
Mean PAP (mmHg)	S1Intensity	0.075	0.646	0.075	0.647
	S2Intensity	0.253	0.116	0.255	0.113
	S3Intensity	0.206	0.202	0.277	0.084
	S4Intensity	0.220	0.185	0.261	0.108
	S1Complexity	− 0.014	0.933	− 0.094	0.565
	S2Complexity	0.300	0.060	0.426	0.006
	S3Strength	0.176	0.278	0.347	0.028
	S4Strength	0.196	0.238	0.070	0.674
PAWP (mmHg)	S1Intensity	− 0.164	0.313	− 0.090	0.579
	S2Intensity	0.038	0.817	0.090	0.583
	S3Intensity	0.276	0.085	0.344	0.030
	S4Intensity	0.104	0.534	0.145	0.379
	S1Complexity	− 0.077	0.636	− 0.178	0.272
	S2Complexity	0.031	0.850	0.083	0.611
	S3Strength	0.499	0.001	0.581	< 0.001
	S4Strength	0.151	0.367	0.065	0.695
PVR (dynes s cm^−5^)	S1Intensity	0.074	0.651	0.115	0.479
	S2Intensity	0.182	0.261	0.193	0.233
	S3Intensity	0.026	0.876	0.124	0.447
	S4Intensity	0.172	0.301	0.233	0.154
	S1Complexity	− 0.073	0.653	− 0.155	0.339
	S2Complexity	0.398	0.011	0.474	0.002
	S3Strength	− 0.063	0.700	0.074	0.652
	S4Strength	0.238	0.150	0.177	0.283
CI (l/min/m^2^)	S1Intensity	0.318	0.049	0.256	0.116
	S2Intensity	0.177	0.281	0.153	0.353
	S3Intensity	0.031	0.852	− 0.041	0.803
	S4Intensity	0.077	0.653	0.109	0.516
	S1Complexity	0.193	0.239	0.347	0.031
	S2Complexity	0.042	0.799	0.046	0.779
	S3Strength	− 0.207	0.207	− 0.298	0.066
	S4Strength	− 0.157	0.354	− 0.054	0.748

*RHC* right heart catheterization, *CABs* cardiac acoustic biomarkers, *mean PAP* mean pulmonary arterial pressure, *PAWP* pulmonary artery wedge pressure, *PVR* pulmonary vascular resistance, *CI* cardiac index

**Table 3:** RHC parameters by type of PH

RHC	Type of PH	Mean	SD	*n*	*p* vs none
Mean PAP (mmHg)	No-PH	16.2	4.1	22	
	Prec-PH	31.2	6.9	5	< 0.001
	Ipc-PH	32.2	5.5	5	< 0.001
	Cpc-PH	40.5	7.3	8	< 0.001
PAWP (mmHg)	No-PH	9.0	3.7	22	
	Prec-PH	9.0	5.5	5	0.982
	Ipc-PH	22.4	4.6	5	< 0.001
	Cpc-PH	19.5	3.6	8	< 0.001
PVR (dynes s cm^−5^)	No-PH	117.6	53.1	22	
	Prec-PH	332.3	134.7	5	< 0.001
	Ipc-PH	157.1	78.0	5	0.415
	Cpc-PH	428.4	160.2	8	< 0.001
CI (l/min/m^2^)	No-PH	2.7	0.8	21	
	Prec-PH	2.6	0.5	5	0.629
	Ipc-PH	2.7	1.0	5	0.975
	Cpc-PH	1.9	0.2	8	0.006

*RHC* right heart catheterization, *mean PAP* mean pulmonary arterial pressure, *PAWP* pulmonary artery wedge pressure, *PVR* pulmonary vascular resistance, *CI* cardiac index, *PH* pulmonary hypertension, *Prec-PH* pre-capillary PH, *Ipc-PH* isolated post-capillary PH, *Cpc-PH* combined pre- and post-capillary PH

**Table 4:** CAB values by type of PH

CAB	Type of PH	3L			*p* vs No-PH	V4			*p* vs No-PH
		Mean	SD	*n*		Mean	SD	*n*	
S1Intensity	No-PH	2.9	3.8	22		2.8	3.7	22	
	Prec-PH	5.0	5.5	5	0.263	4.8	4.5	5	0.290
	Ipc-PH	4.5	3.6	5	0.389	4.3	3.0	5	0.438
	Cpc-PH	2.0	1.6	8	0.535	2.8	3.8	8	0.974
S2Intensity	No-PH	1.4	1.3	22		1.3	1.1	22	
	Prec-PH	3.8	3.3	5	0.044	3.4	2.9	5	0.048
	Ipc-PH	4.4	5.1	5	0.014	4.0	4.6	5	0.013
	Cpc-PH	1.6	1.3	8	0.906	1.7	1.4	8	0.675
S3Intensity	No-PH	0.3	0.3	22		0.2	0.2	22	
	Prec-PH	0.4	0.3	5	0.537	0.5	0.5	5	0.332
	Ipc-PH	0.8	1.1	5	0.033	0.7	1.0	5	0.032
	Cpc-PH	0.4	0.4	8	0.606	0.5	0.5	8	0.211
S4Intensity	No-PH	0.2	0.2	21		0.2	0.2	22	
	Prec-PH	0.5	0.7	5	0.244	0.4	0.5	5	0.144
	Ipc-PH	0.7	1.1	4	0.042	0.5	0.6	4	0.033
	Cpc-PH	0.2	0.2	8	0.951	0.3	0.3	8	0.512
S1Complexity	No-PH	5.4	2.8	22		4.8	2.5	22	
	Prec-PH	4.8	2.1	5	0.696	4.8	1.6	5	0.966
	Ipc-PH	6.0	4.3	5	0.682	4.5	3.0	5	0.799
	Cpc-PH	4.7	2.9	8	0.549	3.8	2.5	8	0.306
S2Complexity	No-PH	2.4	2.0	22		2.1	1.6	22	
	Prec-PH	4.9	3.5	5	0.032	5.3	3.5	5	0.003
	Ipc-PH	2.4	1.8	5	0.994	3.0	1.9	5	0.357
	Cpc-PH	3.8	2.3	8	0.145	3.5	1.9	8	0.083
S3Strength	No-PH	3.8	2.1	22		3.6	2.0	22	
	Prec-PH	3.5	1.8	5	0.744	3.6	2.1	5	0.997
	Ipc-PH	4.8	2.0	5	0.395	4.9	2.1	5	0.222
	Cpc-PH	4.8	2.7	8	0.281	5.4	2.5	8	0.045
S4Strength	No-PH	3.3	1.0	21		3.3	0.8	22	
	Prec-PH	3.4	1.3	5	0.812	3.1	1.4	5	0.749
	Ipc-PH	2.7	1.1	4	0.469	2.8	1.3	4	0.538
	Cpc-PH	4.2	2.5	8	0.127	4.0	2.8	8	0.327

*CABs* cardiac acoustic biomarkers, *PH* pulmonary hypertension, *Prec-PH* pre-capillary PH, *Ipc-PH* isolated post-capillary PH, *Cpc-PH* combined pre- and post-capillary PH

Diagnostic accuracy using CABs for the three types of PH by ROC analysis is shown in Table 5. The AUC of identifying Prec-PH from the other categories by S2Intensity or S2Complexity ranged from 0.674 to 0.720, where sensitivity and specificity ranged from 0.400 to 0.800 and from 0.600 to 1.000, respectively. Moreover, for identification of Ipc-PH by S2Intensity, S3Intensity, and S4Intensity, AUC ranged from 0.657 to 0.807, where sensitivity and specificity varied from 0.800 to 1.000 and from 0.471 to 0.743, respectively. The accuracy at the V4 measurement site was generally consistent with that at 3L. There were no clear improvements of sensitivity and specificity when different CABs were combined to identify Prec-PH and Ipc-PH, other than relatively higher values for NPV than for PPV.

**Table 5:** Diagnostic accuracy of CABs by the type of PH

Type of PH	CAB	Site	AUC	Threshold	Sensitivity	Specificity	PPV	NPV
Prec-PH	S2Intensity	3L	0.674	4.13	0.600	0.829	0.333	0.935
		V4	0.674	3.18	0.600	0.800	0.300	0.933
	S2Complexity	3L	0.697	3.57	0.800	0.600	0.222	0.955
		V4	0.720	8.16	0.400	1.000	1.000	0.921
	Combination	3L	–	–	0.800	0.486	0.174	0.947
		V4	–	–	0.600	0.784	0.273	0.935
Ipc-PH	S2Intensity	3L	0.686	1.60	0.800	0.714	0.286	0.962
		V4	0.669	1.61	0.800	0.714	0.286	0.962
	S3Intensity	3L	0.646	0.21	0.800	0.600	0.222	0.955
		V4	0.657	0.26	0.800	0.600	0.222	0.955
	S4Intensity	3L	0.662	0.10	1.000	0.471	0.182	1.000
		V4	0.807	0.22	1.000	0.743	0.308	1.000
	Combination	3L	–	–	1.000	0.371	0.185	1.000
		V4	–	–	0.800	0.571	0.211	0.952
Cpc-PH	S3Strength	V4	0.742	3.15	1.000	0.469	0.320	1.000

*CABs* cardiac acoustic biomarkers, *PH* pulmonary hypertension, *Prec-PH* pre-capillary PH, *Ipc-PH* isolated post-capillary PH, *Cpc-PH* combined pre- and post-capillary PH, *PPV* positive predictive value, *NPV* negative predictive value

## Discussion

The accuracy of CAB parameters to diagnose PH phenotypes was investigated. Several parameters measured by CABs were significantly correlated with RHC parameters. Previously, Chan et al. also reported significantly higher heart sound complexity in patients with PAH and a positive correlation with mean PAP [23]. In this analysis, significant correlations were observed between the other measurement values of heart sound parameters (intensity and strength) and the hemodynamic parameters (mean PAP, PAWP, and PVR) measured by RHC.

Moreover, significant differences were observed in CABs by the PH phenotypes. Therefore, the CAB values are supportive information indicating abnormal cardiopulmonary function and may be surrogate indicators for RHC parameters. Regarding the accuracy for PH typing of CABs, the AUC ranged from 0.674 to 0.720 for Prec-PH and 0.646 to 0.807 for Ipc-PH, and the AUC was 0.742 for Cpc-PH. These results were comparable to echocardiographic monitoring, where the diagnostic accuracy for typing of PH was reported to range from 0.689 to 0.8 [14–16]. CABs may provide healthcare practitioners an ambulatory method of monitoring PH-LHD patients that is more convenient and less expensive than echocardiography.

**Fig. 2:**
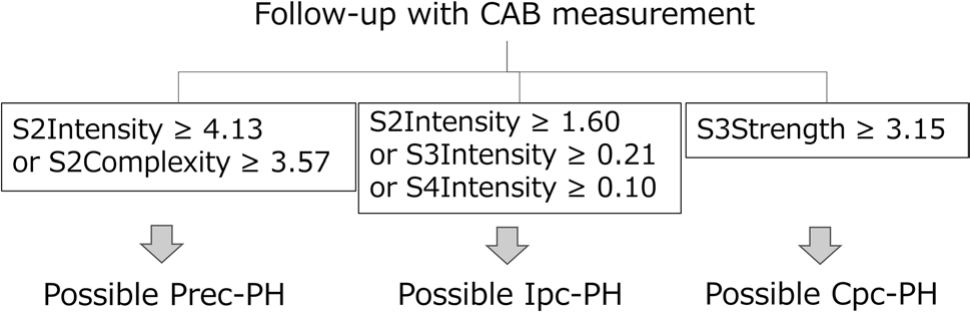
Flowchart for phenotype screening for pulmonary hypertension in the clinical setting. *CABs* cardiac acoustic biomarkers, *PH* pulmonary hypertensions, *Prec-PH* pre-capillary PH, *Ipc-PH* isolated post-capillary PH, *Cpc-PH* combined pre- and post-capillary PH

Since relatively high NPV values were observed in this dataset, CABs may also have potential to identify the No-PH population. In clinical settings, several scenarios using CABs can be implemented. For in-hospital use, CABs are positioned as a screening tool for patients requiring precise examination, such as echocardiographic measurement or RHC. For instance, a patient who has S2Intensity, S3Intensity or S4Intensity greater than or equal to 1.60, 0.21 or 0.10, respectively at 3L site, has suspected Ipc-PH. If a patient shows S2Intensity or S2Complexity greater than or equal to 4.13 or 3.57, respectively at 3L site, these criteria indicate possible Prec-PH. These patients should be referred for echocardiography or RHC (Fig. 2). For Ipc-PH and Cpc-PH screening, S3Intensity and S3Strength showed significant difference compared to No-PH, respectively. Since louder S3 are related to elevation of LVEDP, this correlation may be based on the nature of post-capillary PH due to left heart disease. On the other hand, the screening criterion of Prec-PH are composed of S2-related CABs only. Pulmonic valve component of S2 sound can be affected by load in right ventricle due to pressure elevation occurred at pre-capillary and thus intensity and complexity are thought to be associated.

Since CABs can be easily obtained by non-invasive devices with ECG and PCG sensors, they may also be useful indicators for home tele-monitoring systems to evaluate worsening of heart failure. In such systems, patients can record daily CABs by themselves like blood pressure and weight. Long-term trend of CABs may provide useful information to detect worsening signs prior to an actual HF event and enables physicians to consider early and preventive intervention. This idea is purely conceptual at this point and further studies are needed to evaluate the effectiveness and usability of self-recorded CABs by patients at home.

There are several limitations in this analysis. The sample size was relatively small, and the PH groups were not matched by age and sex. However, there were no significant differences between the groups, and significant relationships were observed between CABs and RHC parameters. Furthermore, the general information regarding ICU inpatients were limited; therefore, the differences of hemodynamic and respiratory condition between inpatients and outpatients were unknown and may have had impact on CABs. Second, echocardiography was not measured simultaneously in this dataset. Therefore, a prospective study with parallel measurement should be performed. Third, this result cannot be directly applied to patients with positive pressure ventilation due to lack of ventilated cases in the present study.

In conclusion, CABs may provide an ambulatory method with home-monitoring friendliness which is more convenient than standard examinations to identify presence of PH and its phenotypes.

## Data Availability

The datasets generated during and/or analyzed during the current study are not publicly available due to confidentiality agreements with research collaborators. Therefore, supporting data can only be made available to researchers subject to a non-disclosure agreement.
